# Development and validation of a depression risk prediction model for middle-aged and elderly adults with sensory impairment: Evidence from the China health and retirement longitudinal study

**DOI:** 10.1371/journal.pone.0332907

**Published:** 2025-09-22

**Authors:** Mengzhen Qin, Mengyuan Qiao, Yuying Dong, Haiyan Wang

**Affiliations:** 1 Shandong Xiehe University, Jinan City, Shandong Province, China; 2 School of Nursing, Henan University of Science and Technology, Luoyang, Henan, China; 3 Medical School, Shihezi University, Shihezi, Xinjiang, China; 4 Xinjiang Emergency Center, People’s Hospital of Xinjiang Uygur Autonomous Region, Urumqi, Xinjiang, China; Shanghai Jiaotong University: Shanghai Jiao Tong University, CHINA

## Abstract

**Objective:**

Compared with those without such impairment, middle-aged and older adults with sensory impairment (SI) demonstrate a greater prevalence and severity of depressive symptoms, significantly affecting their mental health. We aimed to develop and validate a depression risk prediction model for middle-aged and elderly individuals with SI.

**Methods:**

Data from the 2018 China Health and Retirement Longitudinal Study were randomly partitioned into training and validation sets at a 7:3 ratio. Within the training set, least absolute shrinkage and selection operator (LASSO) regression analysis and binary logistic regression were used to identify predictor variables, and a risk prediction column‒line graph was subsequently developed, with depression status among middle-aged and elderly individuals with SI as the dependent variable. Predictive performance of the training and validation sets was assessed via receiver operating characteristic (ROC) curves, calibration plots, and decision curve analysis.

**Results:**

In total, 5308 middle-aged and older adults with SI were included, with 50.1% (n = 2657) developing depression. Multifactorial logistic regression analysis identified several depression predictors, including sex, education level, place of residence, marital status, self-rated health, life satisfaction, pension insurance status, nighttime sleep duration, functional impairment status, and pain (all *P* < 0.05), which were incorporated into a column–line graph that demonstrated good consistency and accuracy. The areas under the ROC curves for the predictive models in the training and validation sets were 0.797 (95% *CI* = 0.783–0.811) and 0.778 (95% *CI* = 0.755–0.800), respectively. The Hosmer–Lemeshow values were *P* = 0.176 and *P* = 0.606 (P > 0.05), and the calibration curves revealed significant agreement between the model and actual observations. ROC and DCA curves indicated good predictive performance for the column‒line graph.

**Conclusion:**

This study presents a reliable, validated, and acceptable predictive model for depression risk in middle-aged and elderly individuals with SI, and the identified predictors have potential applications in public health policy and clinical practice.

## 1 Introduction

With the ageing of the global population, the health of the middle-aged and elderly populations is becoming increasingly important. Among the potential conditions affecting the health of these populations, sensory impairment (SI) (such as visual or hearing impairment), a common geriatric disorder, becomes more prevalent with age [1]. It is estimated that over 1 billion people worldwide are blind or visually impaired [2], and 1.57 billion people suffer from hearing loss [3]. Approximately 13% of adults aged 40–49 years experience some form of hearing loss, whereas nearly 45% of older adults aged 60–69 years suffer from hearing loss, with the prevalence increasing to 90% for those aged 80 years and older [4]. Studies have shown that the negative impact of SI on health outcomes in older adults is often overshadowed by the negative impact of chronic disease and dysfunction [5]. However, the significance of SI as a crucial risk factor for the development of depression in middle-aged and elderly patients should not be overlooked and warrants in-depth exploration.

Depression is highly prevalent in the middle-aged and elderly population and represents the most prominent psychological issue in this population. In China, the prevalence of depression in middle-aged and elderly individuals over 45 years of age exceeds 30% [6]. Research indicates that SI can lead to communication challenges, potentially increasing feelings of loneliness [7], reducing social participation and quality of life [8,9], and triggering anxiety, depression [10], and cognitive decline in older adults. Compared with that in the general elderly population, the prevalence of depression is 2 and 1.27 times greater in visually- and hearing-impaired elderly individuals, respectively [10,11], imposing a considerable economic burden on patients, families, and society. While existing studies have focused primarily on exploring the relationship between SI status and the risk of developing depression, there is a lack of tools for predicting the development of depression in middle-aged and elderly people with SI, hampering effective prevention efforts. Therefore, the aim of this study was to investigate the risk of developing depression in the middle-aged and elderly population with SI via national survey data from the China Health and Retirement Longitudinal Study (CHARLS) by identifying influencing factors, developing and validating a depression risk prediction model for this population, and offering robust support for clinical practice and the formulation of public health policies.

## 2 Materials and methods

### 2.1 Research design and data sources

**Fig 1:**
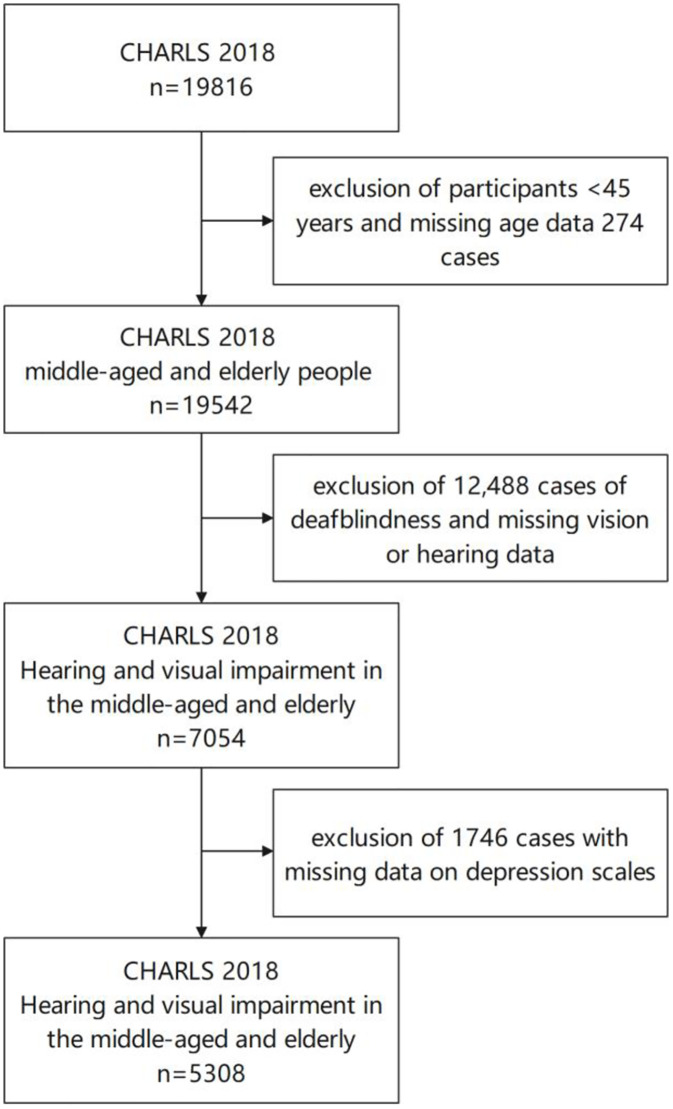
Flowchart of data screening.

The data used for this article were obtained from the CHARLS investigators and are publicly available at https://charls.pku.edu.cn/. The CHARLS is an ongoing longitudinal survey that provides high-quality microdata on households and individuals aged 45 years and older in China With the aim of analysing population ageing issues and promoting interdisciplinary research on ageing. The project received approval from the Biomedical Ethics Committee (IRB00001052–11015) of Peking University in Beijing, China, and our study strictly adhered to the principles outlined in the Declaration of Helsinki; informed consent was obtained from all participants. For this study, we utilized data from the 2018 CHARLS, extracting information on demographic background, health status, functioning status, visual/hearing impairment status, and the Center for Epidemiologic Studies Depression Scale (CES-D10) score. The inclusion criteria were age ≥ 45 years and the presence of visual or hearing impairment. The exclusion criteria were age < 45 years or missing age, visual or hearing status, or depression status data. A total of 5,308 respondents were included in this study. The data screening process is outlined in Fig 1.

### 2.2 Definition of the middle-aged and elderly populations with SI

Middle-aged and older adults were defined as individuals over 45 years of age. Hearing impairment was determined by asking participants questions such as “DA038: Do you ever wear a hearing aid?” and “DA039: Is your hearing very good, good, fair, poor, or very poor (with a hearing aid if you normally use it and without if you normally don’t)?” Participants were considered to have hearing impairment if they answered “yes” to DA038 and/or selected “poor” on DA039 [12]. Visual impairment was assessed by questions such as “DA033: How good is your eyesight for seeing things at a distance, like recognizing a friend from across the street (with glasses or corrective lenses if you wear them)? Would you say your eyesight for seeing things at a distance is excellent, very good, good, fair, or poor?” and “DA034: How good is your eyesight for seeing things up close, like reading ordinary newspaper print (with glasses or corrective lenses if you wear them)? Would you say your eyesight for seeing things up close is excellent, very good, good, fair, or poor?” The participants were considered visually impaired if they selected “poor” for either distance or near vision [13]. The participants were classified as having SI if they had either hearing or visual impairment.

### 2.3 Outcome variables and predictor variables

#### 2.3.1 Assessment of depression.

The dependent variable in this study was the presence of depression, which was determined via the short version of the Center for Epidemiologic Studies Depression Scale (CES-D10) from the CHARLS database. The scale consists of 10 questions with scores ranging from 0 to 3. A score of ≥10 on the CES-D10 indicated depression, whereas a score of <10 indicated no depression. Higher scores indicate more severe depressive symptoms [14].

#### 2.3.2 Demographic factors.

Demographic factors included age, sex (male, female), marital status (married with partner, separated or divorced, widowed, or unmarried), education level (below elementary school, elementary school, middle school, high school, and above), and place of residence (rural, urban).

#### 2.3.3 Health status and behavioural factors.

Health status and behavioural factors included Drinking status, smoking status, chronic disease status (e.g., hypertension, diabetes, heart disease, stroke, dyslipidaemia, memory disorders), sleep patterns, functional impairment status, disability status, and pain status. Dysfunction was assessed by self-reported disability in activities of daily living (ADLs) and instrumental activities of daily living (IADLs). The ADL items included eating, bathing, dressing, getting in and out of bed, toileting, and controlling urination and defecation, whereas the IADL items included housework, meal preparation, shopping, money management, and medication. There were four options for each item, and participants who had no difficulties were considered to have a score of 0; otherwise, a score of 1 was given. Scores from the ADL and IADL items were combined to indicate the level of functional impairment, with scores ranging from 0 to 11, where higher scores indicated higher levels of functional impairment [15]. Disability was determined by asking participants, “Do you have any of the following disabilities?” A “yes” response to any of the questions indicated a disability. Pain was assessed on a scale of 1 to 5 (1 = not at all, 2 = a little, 3 = some, 4 = more, 5 = very much).

#### 2.3.4 Mental health factors.

Mental health factors included self-assessed health and life satisfaction. Self-assessed health was assessed by asking participants, “Do you consider your health to be very good, good, fair, poor, or very poor?”. Life satisfaction was assessed by asking participants “Think about your whole life. How satisfied are you with it? Are you completely satisfied, very satisfied, somewhat satisfied, not very satisfied, or not at all satisfied?”

### 2.4 Statistical analysis

Data from the 2018 CHARLS database were extracted and analysed for this study. The missing data values, which were all less than 20%, were imputed via the Random Forest algorithm. Categorical variables are summarized using frequencies and percentages, whereas continuous variables are presented as the means ± standard deviations or medians with interquartile ranges. Between-group comparisons were conducted via t tests, χ^2^ tests, and nonparametric tests. The data were randomly split into a training set (n = 3716) and a validation set (n = 1592) at a 7:3 ratio. LASSO regression and tenfold cross-validation were applied to the training set to identify the most significant predictors. These predictors were then included in a multifactor logistic regression analysis, and a column‒line graph was created to visualize the risk of developing depression in the middle-aged and elderly population with SI. Receiver operating characteristic (ROC) curves were used to evaluate the model’s discrimination ability. The goodness-of-fit of the model was assessed via the Hosmer–Lemeshow test, and calibration curves were generated. Clinical validity was evaluated via decision curve analysis (DCA). All the statistical analyses were conducted via R software version 4.3.2, and *P* < 0.05 was considered to indicate statistical significance for all the tests.

## 3 Results

### 3.1 Basic information and clinical characteristics of the dataset

The present study included a total of 5308 middle-aged and elderly individuals with SI, comprising 2369 males (43.9%) and 2979 females (56.1%), among whom 2657 participants (50.1%) experienced depression. The study population was randomly divided into training and validation sets at a 7:3 ratio, with 3716 individuals in the training set, of whom 1852 participants (49.8%) developed depression, and 1592 in the validation set, of whom 805 participants (50.6%) developed depression. There was no statistically significant difference in the comparison of clinical data between the training and validation sets (*P* > 0.05), as shown in Supplementary Material Table S1. On the basis of the presence or absence of depression, the samples from the training set were categorized into a depressed group (n = 1864) and a nondepressed group (n = 1852). Univariate analysis revealed that the differences between the two groups were statistically significant (*P* < 0.05) for 18 variables, including sex, education level, place of residence, marital status, Drinking status, smoking status, hypertension status, heart disease status, stroke status, dyslipidaemia status, memory disorder status, and self-rated health status, as shown in Table 1.

**Table 1:** Comparison of the characteristics of the baseline profile in the training set study population.

Variable	Overall (n = 3716)	Nondepressed group (n = 1864)	Depressed group (n = 1852)	Statistic	*P value*
Age, M (Q₁, Q₃)	62.00 (54.00, 69.00)	62.00 (54.00, 69.00)	62.00 (55.00, 69.00)	Z = −0.43	0.670
Sex, n(%)				χ² = 70.37	<0.001
Male	1629 (43.84)	944 (50.64)	685 (36.99)		
Female	2087 (56.16)	920 (49.36)	1167 (63.01)		
Education level, n(%)				χ² = 71.14	<0.001
Below elementary school	1603 (43.14)	682 (36.59)	921 (49.73)		
Primary School	1107 (29.79)	609 (32.67)	498 (26.89)		
Secondary School	682 (18.35)	372 (19.96)	310 (16.74)		
High school and above	324 (8.72)	201 (10.78)	123 (6.64)		
Residence, n(%)				χ² = 52.32	<0.001
Rural	2428 (65.34)	1113 (59.71)	1315 (71.00)		
Urban	1288 (34.66)	751 (40.29)	537 (29.00)		
Marital status, n(%)				χ² = 36.16	<0.001
Married living with partner	2944 (79.22)	1549 (83.10)	1395 (75.32)		
Separated or divorced	283 (7.62)	125 (6.71)	158 (8.53)		
Widowed or unmarried	489 (13.16)	190 (10.19)	299 (16.14)		
Drinking status, n(%)				χ² = 32.24	<0.001
No	2583 (69.51)	1216 (65.24)	1367 (73.81)		
yes	1133 (30.49)	648 (34.76)	485 (26.19)		
Smoking status, n(%)				χ² = 18.80	<0.001
No	2751 (74.03)	1322 (70.92)	1429 (77.16)		
yes	965 (25.97)	542 (29.08)	423 (22.84)		
Hypertension status, n(%)				χ² = 11.47	<0.001
No	2129 (57.29)	1119 (60.03)	1010 (54.54)		
yes	1587 (42.71)	745 (39.97)	842 (45.46)		
Diabetes status, n(%)				χ² = 1.64	0.200
No	3105 (83.56)	1572 (84.33)	1533 (82.78)		
yes	611 (16.44)	292 (15.67)	319 (17.22)		
Heart disease status, n(%)				χ² = 39.07	<0.001
No	2750 (74.00)	1463 (78.49)	1287 (69.49)		
yes	966 (26.00)	401 (21.51)	565 (30.51)		
Stroke status, n(%)				χ² = 12.56	<0.001
No	3403 (91.58)	1737 (93.19)	1666 (89.96)		
yes	313 (8.42)	127 (6.81)	186 (10.04)		
Dyslipidaemia status, n(%)				χ² = 5.57	0.018
No	2750 (74.00)	1411 (75.70)	1339 (72.30)		
yes	966 (26.00)	453 (24.30)	513 (27.70)		
Memory disorder status, n(%)				χ² = 36.14	<0.001
No	3511 (94.48)	1803 (96.73)	1708 (92.22)		
yes	205 (5.52)	61 (3.27)	144 (7.78)		
Self-assessment of health, n(%)				χ² = 390.23	<0.001
very poor	372 (10.01)	81 (4.35)	291 (15.71)		
poor	1131 (30.44)	397 (21.30)	734 (39.63)		
fair	1702 (45.80)	1014 (54.40)	688 (37.15)		
good	279 (7.51)	194 (10.41)	85 (4.59)		
very good	232 (6.24)	178 (9.55)	54 (2.92)		
Life satisfaction, n(%)				χ² = 441.87	<0.001
completely satisfied	161 (4.33)	9 (0.48)	152 (8.21)		
very satisfied	457 (12.30)	82 (4.40)	375 (20.25)		
somewhat satisfied	1999 (53.79)	1039 (55.74)	960 (51.84)		
not very satisfied	945 (25.43)	632 (33.91)	313 (16.90)		
not at all satisfied	154 (4.14)	102 (5.47)	52 (2.81)		
Dysfunction status, M (Q₁, Q₃)	0.00 (0.00, 1.00)	0.00 (0.00, 1.00)	1.00 (0.00, 3.00)	Z = −17.83	<0.001
Medical insurance status, n(%)				χ² = 1.68	0.194
No	109 (2.93)	48 (2.58)	61 (3.29)		
yes	3607 (97.07)	1816 (97.42)	1791 (96.71)		
Pension insurance status, n(%)				χ² = 6.06	0.014
No	1547 (41.63)	739 (39.65)	808 (43.63)		
yes	2169 (58.37)	1125 (60.35)	1044 (56.37)		
Whether you wear glasses or hearing aids, n(%)				χ² = 1.31	0.253
No	2647 (71.23)	1312 (70.39)	1335 (72.08)		
yes	1069 (28.77)	552 (29.61)	517 (27.92)		
Nighttime sleep duration, n(%)				χ² = 162.96	<0.001
<6 h	1501 (40.39)	563 (30.20)	938 (50.65)		
6-8 h	1304 (35.09)	781 (41.90)	523 (28.24)		
≥8 h	911 (24.52)	520 (27.90)	391 (21.11)		
Disability status, n(%)				χ² = 29.84	<0.001
No	3044 (81.92)	1591 (85.35)	1453 (78.46)		
yes	672 (18.08)	273 (14.65)	399 (21.54)		
Pain status, n(%)				χ² = 408.54	<0.001
None	1012 (27.23)	722 (38.73)	290 (15.66)		
A little	1134 (30.52)	627 (33.64)	507 (27.38)		
Somewhat	524 (14.10)	219 (11.75)	305 (16.47)		
Quite a bit	557 (14.99)	162 (8.69)	395 (21.33)		
Very	489 (13.16)	134 (7.19)	355 (19.17)		

Note: Z: Mann-Whitney test, χ²: Chi-square test; M: Median, Q₁: 1st Quartile, Q₃: 3st Quartile.

### 3.2 LASSO regression analysis results

**Fig 2:**
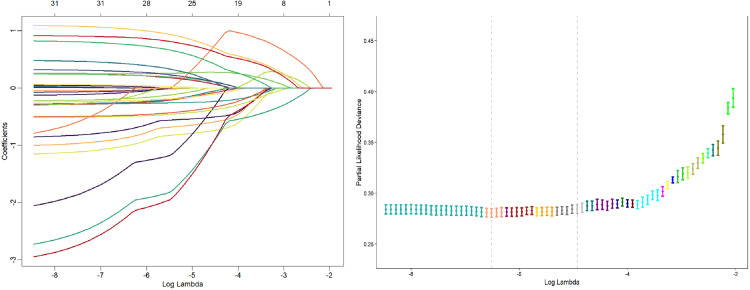
(Left) LASSO Coefficient Path Diagram. (Right) LASSO Regularization Path Diagram.

The independent variables that were statistically significant in the univariate analysis were selected via LASSO regression analysis. To simplify the model, the regularization parameter (λ) corresponding to lambda.1se (lambda = 0.007) was chosen as the optimal λ. A total of 12 variables with nonzero regression coefficients were identified, including sex, education level, place of residence, marital status, memory disorder status, self-assessment of health, life satisfaction, old-age insurance status, sleep quality, dysfunctionality status, disability status, and pain status. The results of the LASSO regression are depicted in Fig 2.

### 3.3 Development of the column–line graph of the predictive model

**Fig 3:**
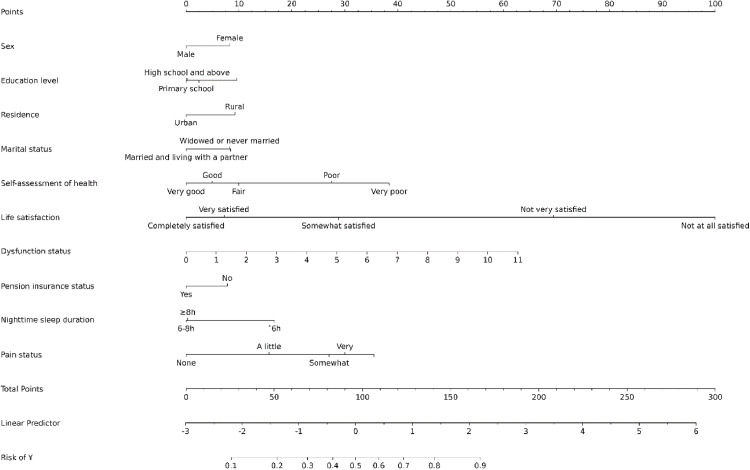
Column–line graph of a depression risk prediction model in middle-aged and elderly individuals with SI.

For further analysis, the 12 variables with regression coefficients not equal to 0, screened by LASSO regression, were included in the multifactorial logistic regression analysis. The results revealed that 10 variables—sex, education level, place of residence, marital status, self-assessed health, life satisfaction, pension insurance status, sleep time at night, functional disability, and pain—were statistically significant (all with *P* < 0.05), as shown in Table 2. A column–line graph was then constructed using these 10 predictor variables to predict the risk of developing depression in middle-aged and elderly individuals with SI in China, as depicted in Fig 3. Each individual’s score for each predictor was determined, and the total score was calculated. The individual probability of occurrence was derived on the basis of the probability corresponding to the total score. A higher total score indicates a greater risk of developing depression in middle-aged and elderly individuals with SI.

**Table 2:** Results of Binary Logistic Regression Analysis.

Variable	β	SE	Z	OR	95% CI	P
Sex						
Male	–					
Female	0.263	0.082	3.194	1.300	1.107–1.528	0.001
Education level						
Below elementary school	–					
Primary School	−0.304	0.097	−3.132	0.738	0.610–0.893	0.002
Secondary School	−0.230	0.113	−2.046	0.794	0.637–0.990	0.041
High school and above	−0.306	0.148	−2.067	0.736	0.551–0.984	0.039
Residence						
Rural	–					
Urban	−0.285	0.084	−3.383	0.752	0.638–0.887	<0.001
Memory disorder status						
No	–					
yes	0.297	0.184	1.613	1.346	0.938–1.930	0.110
Self-assessment of health						
very poor	–					
poor	−0.323	0.158	−2.047	0.724	0.531–0.986	0.041
fair	−0.863	0.156	−5.534	0.423	0.311–0.573	<0.001
good	−1.013	0.205	−4.945	0.363	0.243–0.542	<0.001
very good	−1.164	0.229	−5.082	0.312	0.199–0.489	<0.001
Life satisfaction						
completely satisfied	–					
very satisfied	−0.957	0.381	−2.512	0.384	0.182–0.810	0.012
somewhat satisfied	−2.213	0.362	−6.116	0.110	0.054–0.222	<0.001
not very satisfied	−2.889	0.366	−7.890	0.056	0.027–0.114	<0.001
not at all satisfied	−3.118	0.405	−7.695	0.044	0.020–0.098	<0.001
Pension insurance status						
No	–					
yes	−0.250	0.084	−2.973	0.778	0.660–0.918	0.003
Nighttime sleep duration						
<6 h	–					
6-8 h	−0.518	0.090	−5.749	0.596	0.500–0.711	<0.001
≥8 h	−0.502	0.099	−5.057	0.605	0.498–0.735	<0.001
Disability status						
No	–					
yes	0.055	0.103	0.535	1.057	0.864–1.293	0.600
Pain status						
None	–					
A little	0.487	0.102	4.759	1.628	1.332–1.989	<0.001
Somewhat	0.831	0.127	6.579	2.300	1.792–2.943	<0.001
Quite a bit	1.097	0.133	8.243	2.994	2.307–3.887	<0.001
Very	0.922	0.145	6.345	2.513	1.891–3.341	<0.001
Marital status						
Married living with partner	–					
Separated or divorced	0.258	0.149	1.734	1.294	0.967–1.734	0.083
Widowed or unmarried	0.257	0.122	2.115	1.293	1.019–1.641	0.034
Dysfunction status	0.173	0.025	6.925	1.188	1.132–1.248	<0.001

### 3.4 Validation of a predictive model for depression risk in middle-aged and elderly populations with SI

#### 3.4.1 Discriminative ability of the model.

**Fig 4:**
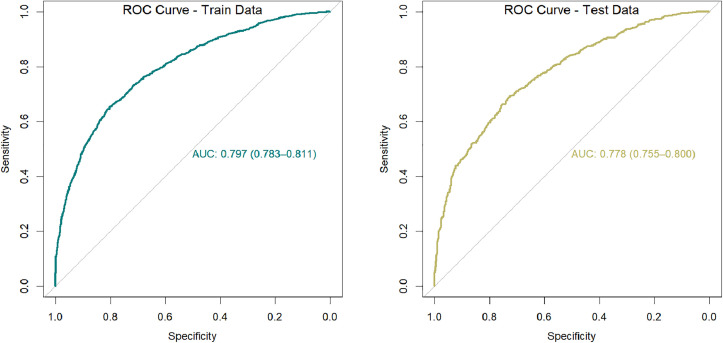
ROC curves for the training and validation sets.

Following the construction of the column–line graph, the discriminative ability of the model in predicting depression risk in middle-aged and elderly individuals with SI was evaluated via the area under the curve (AUC). The ROC curves for the training and validation sets are shown in Fig 4. The predictive model demonstrated an AUC of 0.797 (95% CI = 0.783–0.811), a specificity of 0.802, and a sensitivity of 0.654 in the training set. In the validation set, the AUC was 0.778 (95% CI = 0.755–0.800), with a specificity of 0.729 and a sensitivity of 0.689. These results suggest that the column‒line graph has good discriminatory and predictive ability.

#### 3.4.2 Calibration of the predictive model.

**Fig 5:**
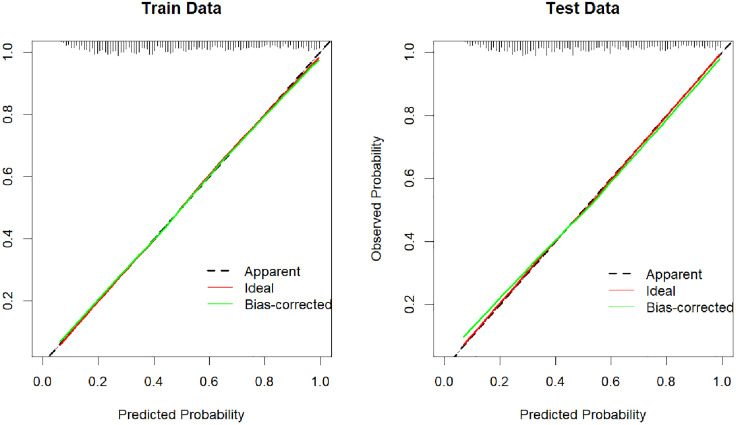
Calibration curves for the training and validation sets.

The Hosmer‒Lemeshow goodness-of-fit test indicated that the model exhibited a good fit (*p* > 0.05) in both the training set (χ^2^ = 11.489, df = 8, p = 0.176) and the validation set (χ^2^ = 6.373, df = 8, p = 0.606). There was a high level of agreement between the predicted probability of depression and the actual probability of depression in both the training and validation sets. The mean absolute error of the calibration curves was 0.005 in the training set and 0.013 in the validation set. The calibration curves for both sets are displayed in Fig 5.

#### 3.4.3 Clinical utility of the predictive models.

**Fig 6:**
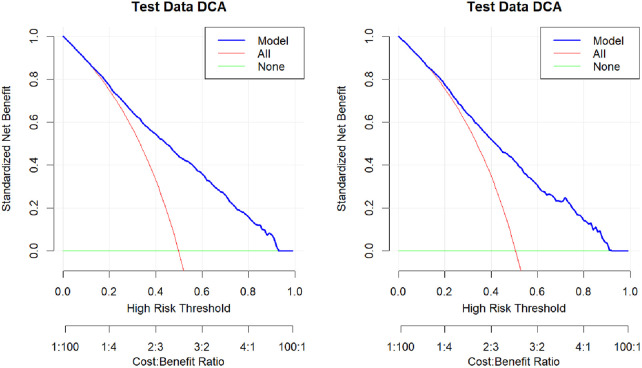
Clinical decision curves for the training and validation sets.

To assess the clinical utility of the risk prediction model, the net clinical benefit was evaluated by plotting a DCA curve, as illustrated in Fig 6. The DCA curves revealed that the column–line graph of the risk prediction model offered clinical benefits to participants within a threshold range of 0.1–0.9, indicating superior net benefit and prediction accuracy and demonstrating good clinical utility.

## 4 Discussion

The study is based on cross-sectional data from 5308 middle-aged and older adults from the 2018 CHARLS database follow-up survey. The data included sociodemographic information, depression status, health status, behavioural factors, and mental health status for each middle-aged and elderly individual with a sensory disorder. On the basis of the 2018 CHARLS database survey, Wu Nianwei et al. [16] reported that the prevalence of depression among middle-aged and older adults in China was 23.61%; however, the results of the present study revealed that the prevalence of depression among middle-aged and older adults with SIs was as high as 50%, which may be related to the fact that visual or hearing impairments aggravate the depressive conditions of middle-aged and older adults.

Sex has long been widely studied as an important factor influencing the onset of depression. Epidemiological studies have consistently shown that the prevalence of depression is significantly greater in women than in men [17–19]. There is also a significant difference in the prevalence of SI between males and females, with female patients significantly outnumbering males [20], a phenomenon that warrants in-depth attention. The present study revealed that the prevalence of depression in middle-aged and elderly individuals with SI was greater in females than in males. This may be related to female psychological characteristics, hormonal changes, social pressure, and other factors. Educational attainment is likewise recognized as an important factor influencing the onset of depression. Studies have shown that well-educated older adults are less likely to develop depression [21], and sociodemographic factors such as education level play important roles in enhancing quality of life and cognitive functioning. In addition, SI is an important factor affecting quality of life and cognitive functioning in older adults [22]. These findings highlight the potential role of education in the prevention and treatment of depression in middle-aged and older populations with SI. A meta-analysis that included 18 studies emphasized [23] that the association between urban and rural residence and the risk of developing depression remained highly significant after adjusting for covariates; the prevalence of depression was greater in rural areas than in urban areas in the Chinese population. In the present study, we reached similar conclusions, possibly because the lack of their children’s companionship and the longer time spent at home make rural empty nesters more likely to feel lonely, which, in turn, leads to the development and exacerbation of depression [24,25].

The present study revealed strong associations between the occurrence of depression and marital status, pension insurance status, and life satisfaction. Middle-aged and older adults who are unmarried (divorced, separated, or widowed) are at a greater risk of developing depression [26]. Research has shown that individuals who experience marital breakdown are more prone to depressive symptoms than those who remain in their marriages are, with this effect being particularly significant among men [27]. These findings highlight the important role of marital status in mental health. Additionally, enrolment in pension insurance has been linked to improved mental health and a reduction in depressive symptoms [28]. Life satisfaction, a key component of subjective well-being, is negatively correlated with the risk of developing depression and anxiety [29]. A cross-sectional study conducted in the U.S. revealed that older adults with moderate or severe hearing impairment had 23% lower levels of subjective well-being and were more likely to experience depressive symptoms even after adjusting for various potential confounding factors [30]. This might be attributed to the fact that older adults with low well-being often experience social isolation and a lack of social support, resulting in decreased life satisfaction, which in turn leads to psychological issues and depressive symptoms.

In addition, the results of this study suggested that self-rated health, nighttime sleep duration, and pain status are also associated with the risk of developing depression. Self-rated health is an important indicator of an individual’s self-perceived health, which is closely related to depression [31]. Liu Yan reported a U-shaped correlation between sleep duration and self-rated health in older adults [32]. The self-rated health of older adults with short sleep durations was significantly lower than that of older adults with normal sleep durations [33], highlighting the importance of improving sleep quality to enhance self-rated health and prevent depression.

Furthermore, we detected a significant association between nighttime sleep duration and depressive symptoms in middle-aged and older populations with SI. A cross-sectional study of 3,840 South African respondents older than 50 years revealed that only short sleep periods of less than 6 hours were associated with self-reported visual impairment [34]. A Chinese study also reported that shorter sleep duration was associated with a greater risk of self-reported visual and hearing impairment [35]. Additionally, compared with non-insomnia status, insomnia status is associated with a twofold risk of developing depression and anxiety [36].

Research evidence also indicates that chronic pain affects sleep quality and that poor sleep quality may contribute to the development or exacerbation of depression [37]. A prospective community-based study revealed that the development of sleep disorders in patients with chronic musculoskeletal pain was associated with a threefold increased risk of developing depression [38]. These findings further support the strong link between depression and insomnia, indicating that a shorter sleep duration can serve as an early warning sign of depression.

Several cross-sectional studies conducted in different countries have shown [39–41] that ADLs are a risk factor for the development of depressive symptoms, potentially having a cumulative effect. In China, a longitudinal study carried out in a Beijing community suggested that impairments in ADLs may increase the risk of developing depressive symptoms in older adults and their spouses [42]. Moreover, findings from the CHARLS database indicate that older adults with depressive symptoms had significantly higher mean ADL scores than did those without depressive symptoms [43]. The present study also confirms that functional impairments, as assessed through ADLs and IADLs, are risk factors for depression in the Chinese middle-aged and elderly population with SIs. Compared with no impairments, even mild visual or hearing impairments can impact the ability of older adults to engage in daily activities and affect their overall quality of life, potentially exacerbating depressive symptoms [44]. Therefore, preventing or reducing impairments in ADLs and IADLs could have a positive effect on the medical care of middle-aged and older adults experiencing depressive symptoms.

Column–line graphs are commonly used in predictive modelling within clinical studies. In this study, a combination of LASSO regression analysis and binary logistic regression analysis was used to identify ten predictors in the training set. These predictors included sex, education level, place of residence, marital status, self-rated health, life satisfaction, pension insurance status, nighttime sleep duration, functional impairment status, and pain status, which were then used to construct a column‒line graph of the risk prediction model. The predictor variables revealed that middle-aged and elderly women with SIs who reside in rural areas, have lower education levels, and live alone are at a higher risk of developing depression, underscoring the need for societal emphasis on this vulnerable group. The model demonstrated an AUC–ROC greater than 0.7 in both the training and validation sets, indicating good discrimination and predictive accuracy. Calibration curves and DCA demonstrated significant consistency between the model’s predictions and actual observations, with high clinical utility for predicting depression. As such, in this study, we present a reliable depression prediction model for middle-aged and elderly Chinese individuals with SIs, with the column–line graph serving as an effective screening tool for depression within this demographic.

This study has several limitations that should be acknowledged. First, while pain is a well-established predictor of depression, our analysis did not account for critical pain-related variables such as location, intensity, or duration. The broad operationalization of pain may limit its clinical interpretability. Furthermore, the inclusion of heterogeneous pain types with distinct pathological characteristics could reduce the model’s ability to accurately identify high-risk populations and inform targeted intervention strategies. Future research should incorporate more refined pain assessment tools to enhance precision. Second, the cross-sectional design precludes causal inference, as it only captures variable associations at a single time point. While the model identifies risk factors correlated with depression, it cannot establish temporal or causative relationships. Longitudinal studies are needed to validate these findings and clarify potential causal pathways. Third, although sensory impairment in this study was defined according to the CHARLS protocol and assessed using validated questionnaire items – an approach that has previously demonstrated significant associations between dual sensory impairment and depressive symptoms [45] – the reliance on self-reported measures rather than objective clinical assessments represents an important limitation. This methodological approach may introduce classification bias due to potential confounding factors such as participants’ cognitive status and social desirability bias. Finally, the model was derived from a Chinese population aged ≥45 years, and its generalizability to other racial, ethnic, or healthcare settings remains uncertain. External validation in diverse cohorts is necessary to confirm its broader applicability.

## 5 Conclusion

In summary, this study identified key predictors of depression in middle-aged and older adults with sensory impairments and developed a predictive model using cross-sectional data analysis. The model exhibited strong discriminative ability and calibration during internal validation. Notably, while the cross-sectional design enables identification of statistical associations between predictors and depression risk, it cannot establish causal temporality. Future studies should leverage longitudinal cohort data and employ advanced analytical approaches, such as cross-lagged panel models or Mendelian randomization, to elucidate the potential causal relationships between sensory impairments, pain, and depression. These findings provide a theoretical foundation for evidence-based early screening of depression in sensory-impaired populations; however, external validation across diverse ethnic, cultural, and healthcare settings is essential to establish generalizability.

## Supporting information

Table S1 Comparison of baseline data between the training set and validation set. (DOCX)
