# Supplementary material for: Development and validation of a depression risk prediction model for middle-aged and elderly adults with sensory impairment: Evidence from the China health and retirement longitudinal study
Source: PLoS One. 2025 Sep 22;20(9):e0332907. doi: 10.1371/journal.pone.0332907 (PMC12453246; doi:10.1371/journal.pone.0332907)
Supplement: Table S1 — (DOCX) [file pone.0332907.s001.docx]

Table S1: Comparison of Baseline Data Between the Training Set and Validation Set

| Variable | Total (n = 5308) | Training Set (n=3716) | Validation Set (n=1592) | Statistic | *P* |
| --- | --- | --- | --- | --- | --- |
| Age, M (Q₁, Q₃) | 62.00 (54.00, 69.00) | 62.00 (54.00, 69.00) | 63.00 (54.00, 69.00) | Z=-0.63 | 0.528 |
| Sex, n(%) |  |  |  | χ²=0.01 | 0.929 |
| Male | 2329 (43.88) | 1629 (43.84) | 700 (43.97) |  |  |
| Female | 2979 (56.12) | 2087 (56.16) | 892 (56.03) |  |  |
| Education level, n(%) |  |  |  | χ²=1.12 | 0.772 |
| Below elementary school | 2310 (43.52) | 1603 (43.14) | 707 (44.41) |  |  |
| Primary School | 1562 (29.43) | 1107 (29.79) | 455 (28.58) |  |  |
| Secondary School | 969 (18.26) | 682 (18.35) | 287 (18.03) |  |  |
| High school and above | 467 (8.80) | 324 (8.72) | 143 (8.98) |  |  |
| Residence, n(%) |  |  |  | χ²=0.20 | 0.654 |
| Rural | 3458 (65.15) | 2428 (65.34) | 1030 (64.70) |  |  |
| Urban | 1850 (34.85) | 1288 (34.66) | 562 (35.30) |  |  |
| Marital status, n(%) |  |  |  | χ²=1.51 | 0.47 |
| Married living with partner | 4191 (78.96) | 2944 (79.22) | 1247 (78.33) |  |  |
| Separated or divorced | 399 (7.52) | 283 (7.62) | 116 (7.29) |  |  |
| Widowed or unmarried | 718 (13.53) | 489 (13.16) | 229 (14.38) |  |  |
| Drinking status, n(%) |  |  |  | χ²=0.63 | 0.427 |
| No | 3707 (69.84) | 2583 (69.51) | 1124 (70.60) |  |  |
| yes | 1601 (30.16) | 1133 (30.49) | 468 (29.40) |  |  |
| Smoking status, n(%) |  |  |  | χ²=0.00 | 0.984 |
| No | 3930 (74.04) | 2751 (74.03) | 1179 (74.06) |  |  |
| yes | 1378 (25.96) | 965 (25.97) | 413 (25.94) |  |  |
| Hypertension status, n(%) |  |  |  | χ²=0.64 | 0.423 |
| No | 3060 (57.65) | 2129 (57.29) | 931 (58.48) |  |  |
| yes | 2248 (42.35) | 1587 (42.71) | 661 (41.52) |  |  |
| Diabetes status, n(%) |  |  |  | χ²=1.55 | 0.214 |
| No | 4457 (83.97) | 3105 (83.56) | 1352 (84.92) |  |  |
| yes | 851 (16.03) | 611 (16.44) | 240 (15.08) |  |  |
| Heart disease status, n(%) |  |  |  | χ²=0.91 | 0.34 |
| No | 3948 (74.38) | 2750 (74.00) | 1198 (75.25) |  |  |
| yes | 1360 (25.62) | 966 (26.00) | 394 (24.75) |  |  |
| Stroke status, n(%) |  |  |  | χ²=2.39 | 0.122 |
| No | 4840 (91.18) | 3403 (91.58) | 1437 (90.26) |  |  |
| yes | 468 (8.82) | 313 (8.42) | 155 (9.74) |  |  |
| Dyslipidaemia status, n(%) |  |  |  | χ²=0.01 | 0.918 |
| No | 3926 (73.96) | 2750 (74.00) | 1176 (73.87) |  |  |
| yes | 1382 (26.04) | 966 (26.00) | 416 (26.13) |  |  |
| Memory disorder status, n(%) |  |  |  | χ²=1.02 | 0.311 |
| No | 5026 (94.69) | 3511 (94.48) | 1515 (95.16) |  |  |
| yes | 282 (5.31) | 205 (5.52) | 77 (4.84) |  |  |
| Self-assessment of health, n(%) |  |  |  | χ²=2.90 | 0.575 |
| very poor | 522 (9.83) | 372 (10.01) | 150 (9.42) |  |  |
| poor | 1640 (30.90) | 1131 (30.44) | 509 (31.97) |  |  |
| fair | 2403 (45.27) | 1702 (45.80) | 701 (44.03) |  |  |
| good | 411 (7.74) | 279 (7.51) | 132 (8.29) |  |  |
| very good | 332 (6.25) | 232 (6.24) | 100 (6.28) |  |  |
| Life satisfaction, n(%) |  |  |  | χ²=5.59 | 0.232 |
| completely satisfied | 238 (4.48) | 161 (4.33) | 77 (4.84) |  |  |
| very satisfied | 619 (11.66) | 457 (12.30) | 162 (10.18) |  |  |
| somewhat satisfied | 2876 (54.18) | 1999 (53.79) | 877 (55.09) |  |  |
| not very satisfied | 1359 (25.60) | 945 (25.43) | 414 (26.01) |  |  |
| not at all satisfied | 216 (4.07) | 154 (4.14) | 62 (3.89) |  |  |
| Dysfunction status, M (Q₁, Q₃) | 0.00 (0.00, 1.00) | 0.00 (0.00, 1.00) | 0.00 (0.00, 2.00) | Z=-0.81 | 0.419 |
| Medical insurance status, n(%) |  |  |  | χ²=0.42 | 0.517 |
| No | 161 (3.03) | 109 (2.93) | 52 (3.27) |  |  |
| yes | 5147 (96.97) | 3607 (97.07) | 1540 (96.73) |  |  |
| Pension insurance status, n(%) |  |  |  | χ²=3.33 | 0.068 |
| No | 2167 (40.83) | 1547 (41.63) | 620 (38.94) |  |  |
| yes | 3141 (59.17) | 2169 (58.37) | 972 (61.06) |  |  |
| Whether you wear glasses or hearing aids, n(%) |  |  |  | χ²=2.31 | 0.128 |
| No | 3748 (70.61) | 2647 (71.23) | 1101 (69.16) |  |  |
| yes | 1560 (29.39) | 1069 (28.77) | 491 (30.84) |  |  |
| Nighttime sleep duration, n(%) |  |  |  | χ²=4.90 | 0.087 |
| <6 h | 2155 (40.60) | 1501 (40.39) | 654 (41.08) |  |  |
| 6-8 h | 1895 (35.70) | 1304 (35.09) | 591 (37.12) |  |  |
| ≥8 h | 1258 (23.70) | 911 (24.52) | 347 (21.80) |  |  |
| Disability status, n(%) |  |  |  | χ²=0.07 | 0.789 |
| No | 4353 (82.01) | 3044 (81.92) | 1309 (82.22) |  |  |
| yes | 955 (17.99) | 672 (18.08) | 283 (17.78) |  |  |
| Pain status, n(%) |  |  |  | χ²=1.65 | 0.8 |
| None | 1433 (27.00) | 1012 (27.23) | 421 (26.44) |  |  |
| A little | 1605 (30.24) | 1134 (30.52) | 471 (29.59) |  |  |
| Somewhat | 750 (14.13) | 524 (14.10) | 226 (14.20) |  |  |
| Quite a bit | 807 (15.20) | 557 (14.99) | 250 (15.70) |  |  |
| Very | 713 (13.43) | 489 (13.16) | 224 (14.07) |  |  |
| Depressed status, n(%) |  |  |  | χ²=0.24 | 0.627 |
| No | 2651 (49.94) | 1864 (50.16) | 787 (49.43) |  |  |
| yes | 2657 (50.06) | 1852 (49.84) | 805 (50.57) |  |  |

Note: Z: Mann-Whitney test, χ²: Chi-square test; M: Median, Q₁: 1st Quartile, Q₃: 3st Quartile
